# Parental concerns of allergy or hypersensitivity and the infant’s diet

**DOI:** 10.1002/nop2.195

**Published:** 2018-08-19

**Authors:** Bettina Holmberg Fagerlund, Sølvi Helseth, Lene Frost Andersen, Milada Cvancarova Småstuen, Kari Glavin

**Affiliations:** ^1^ Department of Nursing and Health Promotion, Faculty of Health Sciences OsloMet – Oslo Metropolitan University Oslo Norway; ^2^ Department of Nutrition, Institute of Basic Medical Sciences, Faculty of Medicine University of Oslo Oslo Norway; ^3^ Department of Health VID Specialized University Oslo Norway

**Keywords:** allergy, child health centre, diet, food, hypersensitivity, infant, parent, toddler

## Abstract

**Aim:**

To investigate a possible association between children's current diet and parents’ reported avoidance of appropriate foods in the child's diet at the age of 10 months, due to fears of allergic or hypersensitivity reactions.

**Design:**

A cross‐sectional study.

**Methods:**

In 10 randomly selected municipalities willing to participate, 686 children represented by their parents visiting the child health centre were enrolled in the study. From January 2015–January 2017, 440 (64%) parents completed a semi‐quantitative food frequency questionnaire concerning their child.

**Results:**

Thirty‐four percent of parents reported that they avoided introducing some food items due to fears of allergy or hypersensitivity in their child. A statistically significant relationship emerged between this reporting and parents wanting more information about food for infants and toddlers. However, the reported fear was not associated with dietary restrictions regarding actual feeding of the child.

## 1 INTRODUCTION

The World Health Organization (2017) currently recommends exclusive breastfeeding for the first 6 months of life, followed by introduction of nutritionally adequate and safe complementary foods together with continued breastfeeding up to 2 years and beyond. An apparent increase in food allergy has resulted in reconsidering prevention strategies aimed at the infant's diet (Du Toit, Foong, & Lack, 2016; Gupta & Sicherer, 2017). Recent research indicates that early introduction of potential allergens in the child's diet (Du Toit et al., 2016; Netting et al., 2017), around 4 months of age while the infant continues to breastfeed might protect against developing food allergies (Smith & Becker, 2016). Whether the age of introduction of complementary food as a means of allergy prevention should be 4 or 6 months has not been established (Abrams, Greenhawt, Fleischer, & Chan, 2017; Smith & Becker, 2016). Accordingly, a compromise so far seems to recommend solid food introduction “at around 6 months but not before 4 months,” as in Australasian guidelines (Abrams et al., 2017). A similar recommendation appears in the Norwegian authorities’ guidelines for advice from child health centres (CHCs) on infant feeding practices (Norwegian Directorate of Health, 2016, 2017). Almost all parents and their under school‐aged children use these municipal CHCs (Statistics Norway, 2016). They provide extensive, universally available preventive health care on a voluntary basis, free of charge (Norwegian Directorate of Health, 2017).

As the prevalence of food allergy may be challenging to determine because adverse food reactions may occur for various reasons (Rona et al., 2007), it is not surprising that the public might use the term “allergy” to describe any adverse response to foods (Sicherer, 2011). Food allergy is a hypersensitivity reaction caused by a specific immune response (Johansson et al., 2004; Venter et al., 2006), estimated to affect nearly 5% of adults and 8% of children (Sicherer & Sampson, 2014). When it comes to hypersensitivity, previously referred to as intolerance (Johansson et al., 2004), the prevalence of food hypersensitivities perceived by the parents only in children aged 1–4 years in south‐eastern Finland was 21% (Pyrhönen, Näyhä, Kaila, Hiltunen, & Läärä, 2009). Corresponding, a study from the UK reported a cumulative incidence of 26% for parentally perceived food hypersensitivity by the child's first year and 34% by the child's third year (Venter et al., 2008).

Food allergy is a serious health issue (Longo, Berti, Burks, Krauss, & Barbi, 2013) and may even be life threatening (Boye, 2012; Longo et al., 2013). In 90% of cases, food allergy is induced by peanuts, cow's milk, hen's eggs, nuts, soybeans, fish, crustaceans and shellfish (Boye, 2012). Avoidance of allergens should be based on clearly defined criteria and thus “avoidance and fear of all” is not appropriate as a strategy in allergy management (Haahtela, von Hertzen, Mäkelä, & Hannuksela, 2008).A previous study by Ilmonen, Isolauri, and Laitinen (2012) among nurses at CHCs in Finland indicated that 20% of the nurses gave incorrect advice on food avoidance as a preventive measure to prevent food allergy.

We aimed to investigate whether there was an association between children's current diet and parents’ reporting of avoiding appropriate foods when feeding their child at the age of 10 months, because they were afraid that the child might react with allergy or hypersensitivity. To our knowledge and based on a literature review, there are no previous studies on this topic.

## 2 METHODS

### 2.1 Design

This cross‐sectional study reports on baseline data in a Cluster Randomized Controlled Trial, registered in ClinicalTrials.gov, Identifier: NCT02266953. At baseline, before the child's 10 month consultation, the parents answered a semi‐quantitative food frequency questionnaire (SFFQ) on behalf of their child.

### 2.2 Setting and sample

In total, there were 139 municipalities consisting of all Norwegian municipalities with more than 100 births in 2012, except those in the three northernmost counties. Of these, we selected 10 municipalities that fulfilled the inclusion criteria of implementing the healthcare programme in a way consistent with the authorities’ regulations regarding timing and number of consultations (Norwegian Directorate of Health, 2004) and were willing to participate. All parents in these 10 municipalities who visited the CHC received oral and written information about the research project from their public health nurse (PHN). The only exclusion criterion was insufficient Norwegian skills to understand written information about the study.

### 2.3 Ethical considerations

The participating parents gave their written consent to the PHN at the CHC when the child was aged 5–6 months. Participation was related to the oldest child if the child was a twin or triplet. Participation was voluntary, and the participant could withdraw without giving a reason. All data have been treated as confidential. Participant anonymity was guaranteed. The study was approved by the Regional Committees for Medical and Health Research Ethics (REC), Ref.nr. 2014/726.

### 2.4 Data collection

#### 2.4.1 The semi‐quantitative food frequency questionnaire

The SFFQ in the present study was designed to investigate feeding practices of 10‐month‐old infants retrospectively from birth. The SFFQ was a revised version of a validated and standardized SFFQ (Kristiansen, Lande, Øverby, & Andersen, 2010) developed for a national dietary survey among 12‐month‐old infants in Norway in 2007 (Spedkost [Infant Diet] 2006–2007). The revisions made for the present study were updates regarding applicable food items on the market. The revisions included new types of formula milk for infants, a new type of children's yoghurt replacing an older type, baby porridge containing milk that replaced older types without milk, a soft cheese product for children that replaced an old type, updated designations of margarines and exclusion of an industrial baby food product that was no longer on sale. Questions regarding the parents’ use of organic food and spinach as an alternative under vegetables were removed in the current SFFQ. The weight and length of the child at 6 months, the mother's use of snuff and the mother's country of origin were questions added in the present SFFQ. Apart from this, the SFFQ consisted of questions as described in detail by Kristiansen et al. (2010).

#### 2.4.2 Completion of semi‐quantitative food frequency questionnaires

Parents were recruited continuously from 5 January 2015 ‐ 31 January 2017. The parents who consented to participate received a paper format SFFQ by postal mail when their child was approximately 8.5 months old. Enclosed with the SFFQ was written information about the survey and a reply envelope. One of the authors administered the distribution of the SFFQs. The parents were asked to complete and return the SFFQ just before the child's 10 month consultation at the CHC. Before the deadline, parents usually received a telephone call to remind them about the questionnaire. Completion of the SFFQ was estimated to take about 40 min.

### 2.5 Data analysis

#### 2.5.1 Statistical analysis

Continuous data were described with median and range, categorical data with counts and percentages. Crude associations between pairs of categorical data were assessed using Chi‐square tests. Possible differences between groups regarding continuous variables were analysed using *t* tests and the Mann‐Whitney‐Wilcoxon test if not normally distributed. To correct for multiple testing, *p* < 0.01 was considered statistically significant. All statistical analyses were performed using *IBM SPSS Statistics for Windows ®, Version 24.0., IBM Corporation®*.

#### 2.5.2 Nutrient calculations

Daily intake of energy, nutrients and food groups was computed using a food database in a software diet calculation program, *KBS [= KostBeregningsSystemet]* in Norwegian, version 7.3, developed at the Department of Nutrition, University of Oslo. The relevant food database is mainly based on a version of the official Norwegian food composition table (Kristiansen, Laugsand Lillegaard, & Andersen, 2013; Norwegian Food Safety Authority, 2017). In the present SFFQ the food database AE‐10 was used, based on the official Norwegian food composition table of 2006.

#### 2.5.3 Categories of parents based on the aim of the study

The parents were divided into two groups, the avoidance and non‐avoidance group, based on their response to the SFFQ. The *avoidance group* consisted of parents who reported that they avoided giving appropriate foods to their child because of their fear that the child would react with allergy or hypersensitivity. Consequently, parents who did not report such actions were categorized in the *non‐avoidance group*.

## 3 RESULTS

### 3.1 Participant characteristics

**Figure 1:**
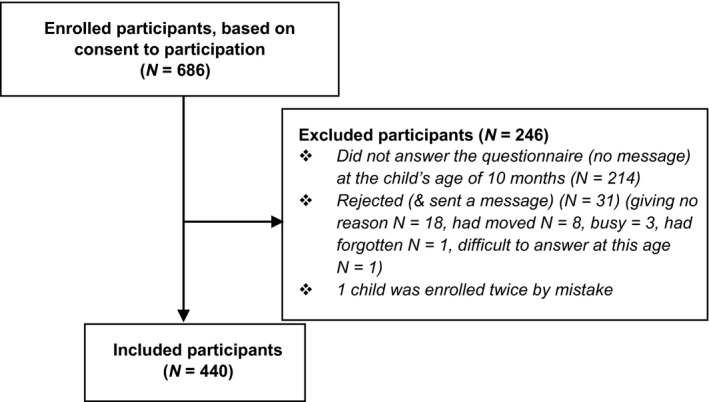
Flowchart of the process including participants and reasons for exclusion

Figure 1 shows the process of including participants. There is no data on how many parents at the CHCs were initially asked about participation in the study. In total, 686 children represented by consenting parents were enrolled in the study. SFFQs were completed for 440 children (64% response rate). Characteristics of the infants (*N* = 440) are presented in Table 1 and of the parents in Table 2. There is a slight overrepresentation of boys among the infants in the sample (Statistics Norway, 2017) (Table 1).

**Table 1:** Characteristics of infants (*N* = 440)

Characteristic	Value
Gender, *n* (%)	
Females/males	213 (48.4)/227 (51.6)
Age (months)^a^, median [range]	9.9 (7.8–11.5)
Birth weight (g), median [range]	3,545 (1075–4070)
Missing (*n*)	19
Birth length (cm), median [range]	50 (37–56)
Missing (*n*)	26
Gestational age at birth (weeks), *n* (%)	
≥38	388 (88.2)
<38	51 (11.6)
Missing	1 (0.2)
Breastfed, *n* (%)	
Yes, currently	232 (52.7)
No, but earlier	194 (44.1)
No, never	11 (2.5)
Missing	3 (0.7)

a at the time of completing the food frequency questionnaire.

**Table 2:** Characteristics of the mothers and the education level of the fathers

Characteristic	Value
Mothers (*N* = 440)	
Age (years), median [range]	30 (18–46)
Marital status, *n* (%)	
Married	189 (43)
Cohabitant	235 (53.4)
Not married/cohabitant	13 (3.0)
Missing	3 (0.7)
Country of origin, *n* (%)	
Norway	401 (91.1)
Rest of Europe	28 (6.4)
Outside Europe	11 (2.5)
Working outside home^a^, *n* (%)	
Yes	149 (33.9)
No	290 (65.9)
Missing	1 (0.2)
Smoking, *n* (%)	
Yes, daily	10 (2.3)
Yes, occasionally	11 (2.5)
No	419 (95.2)
Use of snuff, *n* (%)	
Yes, daily	22 (5.0)
Yes, occasionally	8 (1.8)
No	410 (93.2)
Number of children, *n* (%)	
1	195 (44.3)
2	170 (38.6)
3	58 (13.2)
≥4	16 (3.6)
Missing	1 (0.2)
Education, *n* (%)	
Below upper secondary education	10 (2.3)
Upper secondary education^b^	118 (26.8)
Higher education, short	194 (44.1)
Higher education, long	117 (26.6)
Missing	1 (0.2)
Fathers (*N* = 440)	
Education, *n* (%)	
Below upper secondary education	12 (2.7)
Upper secondary education^b^	221 (50.2)
Higher education, short	120 (27.3)
Higher education, long	76 (17.3)
Missing	8 (1.8)

^a^at the time of completion of the food frequency questionnaire. ^b^Tertiary vocational education is included.

Both mothers and fathers in our sample had higher levels of education than the general population (Statistics Norway, 2017) (Table 3). The proportion of mothers with a country of origin other than Norway is lower in our study compared with the general Norwegian population (Statistics Norway, 2017).

**Table 3:** Length of education in the study sample in comparison to population data from Statistics Norway, (2017)

Mother's education level	Value, *N* (%)	General population, 30–34 year‐old women, (%)
Below upper secondary education	10 (2.3)	17.2
Upper secondary education^a^	118 (26.8)	24.9
Higher education, short	194 (44.1)	37.5
Higher education, long	117 (26.6)	20.4

Father's education level	Value, *n* (%)	General population, 30–34 year old men, (%)
Below upper secondary education	12 (2.7)	23.1
Upper secondary education^a^	221 (50.2)	37.2
Higher education, short	120 (27.3)	23.0
Higher education, long	76 (17.3)	16.8

a Tertiary vocational education is included.

### 3.2 Parent‐reported food avoidance, food allergy and hypersensitivity

The parent‐reported food avoidance was categorized into two groups. Thirty‐four percent (*N* = 151) reported that they avoided giving appropriate food to their child because of their fear that their child might react with allergy or hypersensitivity (hereafter called the avoidance group) (Table 4). The proportion of parents reporting that their child had nutritional problems related to food allergy or food hypersensitivity was very small — only 4.1% (Table 4).

**Table 4:** Parental reporting related to the infant's allergy or hypersensitivity

Question	Value *n* (%)		
	Yes	No	Missing
Is there any applicable food that you avoid offering to your child because you are afraid that your child might react with allergy or hypersensitivity?	151 (34.2)	278 (63.2)	11 (2.5)
Does your child have problems with food and feeding related to food allergy or food hypersensitivity?	18 (4.1)	422 (95.9)	–

There are no statistically significant differences regarding demographic variables of the parents and the family between the two groups, either avoiding giving foods to the child or not (Table 5). However, a significantly higher proportion (68.9%) of parents in the avoidance group wanted more information about food for infants and toddlers than in the non‐avoidance group (46.0%) (Table 5).

**Table 5:** Associations between selected variables and parental avoidance or non‐avoidance of offering certain food because they are afraid the child might react with allergy or hypersensitivity

Question	Do you avoid offering applicable food because you are afraid the child might react with allergy or hypersensitivity?		
Variable	Yes‐mothers, (*N* = 151)	No‐mothers, (*N* = 278)	*p*‐value^*^
	*N* (%)	*N* (%)	
Mother's education level			0.66^a^
Upper secondary education and below^c^	44 (29.1)	80 (28.8)	
Higher education, short	70 (46.4)	119 (42.8)	
Higher education, long	37 (24.5)	79 (28.4)	
Mother's age (years), mean	30.4	31.1	0.15^b^
Number of children			0.56^a^
1	69 (45.7)	122 (44.0)	
2	58 (38.4)	106 (38.3)	
3	21 (13.9)	36 (13.0)	
4	3 (2.0)	13 (4.7)	
Wish for more information about food for infants and toddlers			<0.001^a^
Yes	104 (68.9)	128 (46.0)	
No	41 (27.2)	128 (46.0)	
Don't know	6 (4.0)	22 (7.9)	

	Yes‐fathers, (*N* = 147)	No‐fathers, (*N* = 272)	
	*N* (%)	*N* (%)	
Father's education level			0.27^a^
Upper secondary education and below^c^	84 (57.1)	142 (52.2)	
Higher education, short	34 (23.1)	83 (30.5)	
Higher education, long	29 (19.7)	47 (17.3)	

^a^Pearson's Chi‐Square Test. ^b^T test for equality of means; equal variances assumed. ^c^Tertiary vocational education is included. ^d^ *p*‐values <0.01 considered as statistically significant.

There was no difference between infant food intake in the avoidance group and the non‐avoidance group. Significantly less cheese (*p* < 0.001) was consumed among the children in the sample (*N* = 18) who had problems with food and feeding related to food allergy or food hypersensitivity compared with the rest of the sample (*N* = 422). All other differences between the groups of children with or without problems related to food allergy or food hypersensitivity were registered non‐significant (*p* > 0.01) regarding consumed amounts of food items.

## 4 DISCUSSION

Thirty‐four percent of parents in this study reported that they avoided introducing some food items due to *fears* of allergy or hypersensitivity in their child. However, this fear did not seem to be associated with dietary restrictions regarding the child. The findings in our study showed no differences in the food consumed by the child, aged 10 months, between the avoidance group and the non‐avoidance group. A previous study reports that parents’ *suspicions* of their child having allergy could cause a delay in the introduction of complementary feeding (Niinivirta, Isolauri, Nermes, & Laitinen, 2014). A review article found that the entity “food allergy” included plenty of imagined allergy and accordingly unnecessary avoidance of foods among children was too high (Haahtela et al., 2008). Regarding our study sample, this seems no challenge because of the similar food consumed among infants in the avoidance and non‐avoidance group.

In our study, 71% of the mothers had more than 15 years of education. No differences in the mothers’ educational level, the age of the mothers or the number of siblings were observed in the avoidance group and the non‐avoidance group. In contrast, Eggesbø, Botten, and Stigum (2001) presented, on the basis of Oslo Birth Cohort 1992–1993 (Nafstad, Jaakkola, Hagen, Botten, & Kongerud, 1996), a significant relation between higher maternal education, lower maternal age and a groundless restricted diet for the child. The proportion of mothers with more than 15 years of education was lower in this study compared with ours (53% vs. 71%) (Nafstad et al., 1996). A population‐based cross‐sectional survey among children in south‐eastern Finland revealed that food allergies diagnosed by a physician were less common among children having one or more older siblings than when the index child was the oldest or the only child (Pyrhönen et al., 2009). Further, it has been indicated that an unwarranted diet for a child because of parental suspicions of allergy is significantly related to a child having siblings (Eggesbø et al., 2001).

Our study's findings revealed that 4.1% of parents report that their child has dietary problems related to food allergy or food hypersensitivity. We do not have information on whether this is based on clinically diagnosed or exclusively parental assumption of food allergy and food hypersensitivity. A large study in the UK based on a whole population birth cohort (*N* = 969) found that the cumulative incidence of parentally perceived food hypersensitivity in their child aged 12 months was 25.8%. Of these children, only 14% were objectively diagnosed with food hypersensitivity by means of an open food challenge and 6% were diagnosed with food hypersensitivity by means of a double‐blind, placebo‐controlled food challenge (Venter et al., 2006). Further, a previous literature review reported a proportion of 8% of objectively assessed food allergy in children (Sicherer & Sampson, 2014). Thus, the corresponding percentage in our study is considerably lower than indicated in numerous studies suggesting an increase in food allergy, although these numbers should be treated with caution due to methodological concerns (Sicherer, 2011).

Our data revealed a significant association between parents who wanted more information about food for infants and toddlers and their reported avoidance of giving appropriate food to their child because of fears of allergy or hypersensitivity. Our study thus suggests that parents with concerns related to their infant's food and feeding practices are looking for trustworthy information about child feeding. This is to be expected because during infancy children are completely dependent on their parents’ understanding, effort and practices regarding food and feeding their child. Good parental understanding of their infant's nutritional requirements is essential to make the right decisions for their child (Hobbie, Baker, & Bayerl, 2000).

One‐third of the parents in our study reported a concern for allergy or hypersensitivity in their child in relation to the child's diet. Sixty‐nine percent of these parents wanted more information about food for infants and toddlers. This underlines a need to examine specifically the information delivery related to these themes in the CHC. Further research should investigate what kind of information about food and feeding practices the parents receive and its relation to parental needs and expectations.

### 4.1 Strength and limitations

A relatively large response rate in terms of the type of study, in relation to the number of parents who consented to participate, represents a strength in this study. Participants who were almost exclusively of non‐immigrant background and had a higher level of education than the general Norwegian population might limit the generalizability of our findings. However, despite an overrepresentation of participants with higher education, all levels of education are represented among the participating parents. Due to the study design, causality cannot be inferred. The unknown response rate, based on how many participants were initially invited to participate in the study, also limits generalizability of the results.

## 5 CONCLUSION

This study's focus on parental fears of allergy or hypersensitivity in their infant associated with the infant's diet has rarely been addressed previously. The study findings show that the infant's diet seems unaffected by parents’ concerns regarding food allergy and hypersensitivity in their child. A significant association was revealed between parents reporting avoidance of giving their infant certain foods because of fears of allergy or hypersensitivity in their child and the parents’ wish for more information about food for infants and toddlers. This should be considered in service provision in the CHCs.

## 6 TRIAL REGISTRATION

This cross‐sectional study reports on baseline data in a Cluster Randomized Controlled Trial on the use of a communication tool about diet at the child health centre, registered in ClinicalTrials.gov, Identifier: NCT02266953.

## CONFLICT OF INTEREST

The authors declare that they have no conflict of interest.

## AUTHOR CONTRIBUTIONS

BHF, KG, SH, LFA, MCS: designed the study. BHF: facilitated for collecting data. BHF: collected the data. BHF, MCS, LFA, KG, SH: contributed to data analysis. BHF, KG, SH, LFA, MCS: contributed to preparing the manuscript. BHF, KG, SH, LFA, MCS: read and approved the final manuscript.

All authors have agreed on the final version and meet at least one of the following criteria [recommended by the ICMJE (https://www.icmje.org/recommendations/)]:

- substantial contributions to conception and design, acquisition of data or analysis and interpretation of data;
- drafting the article or revising it critically for important intellectual content.
